# Research on the formation mechanism of information cocoon and individual differences among researchers based on information ecology theory

**DOI:** 10.3389/fpsyg.2022.1055798

**Published:** 2022-12-20

**Authors:** Xiaofang Yuan, Chunyun Wang

**Affiliations:** Management School, Xi’an University of Science and Technology, Xi'an, China

**Keywords:** information cocoon, formation mechanism, scientific researchers, information ecology theory, individual differences

## Abstract

**Introduction:**

Choosing to only retrieve and read academic information related to their own research field is conducive to researchers’ in-depth understanding of their own research issues, and also reduces the pressure of researchers’ information retrieval, but may bring about the effect of information cocoon.

**Methods:**

Based on the information ecology theory, a theoretical model is built from the aspects of information people, information, information environment and information technology, and relevant data is collected through questionnaires to verify assumptions. To explore the formation path of information cocoon for scientific researchers and the differences between different types of scientific researchers.

**Results:**

Different gender, education background, identity, age and team size of scientific researchers will lead to different dimensions in the process of producing information cocoon; Community influence, information homogeneity and value identification are important factors influencing the formation of information cocoon room for scientific researchers; Community influence and information homogeneity positively affect value identification.

**Discussion:**

In the future research work, researchers should pay attention to the influence of information homogeneity, community influence and value identification.

## 1. Introduction

The explosion of information in the era of big data and the overload of information make people’s information behavior show characteristics such as increased network dependence, “fragmentation,” and selective exposure. Digital technology allows us to filter information, but it also leads us to exchange information with like-minded people. In addition, because the limited rationality of human beings can not cope with the amount of information on the Internet, we are faced with the problem of cognitive dissonance. We try to solve these problems by ignoring the views that are too different from our own (Gossart, 2014). With the development of information technology, digital academic information also shows the characteristics of massive and multiple. Researchers often search and read-only academic information related to their research fields to find practical information more quickly and effectively. This information behavior is conducive to researchers’ in-depth understanding of their research problems and reduces the pressure of information retrieval for researchers. However, focusing only on homogeneous information for a long time may bring about problems such as information narrowing and group polarization, eventually creating information cocoons. This will be detrimental to cross-disciplinary scholarly communication and harms the development of individual researchers and even the progress of related research fields. Fan et al. pointed out that information cocoons can deepen users’ inherent biases, leading to their self-perception bias and irrational inflation, making them prone to form radical and extreme views, statements, or behaviors (Fan and Sun, 2019). The extreme consequence of academic information cocooning leads to the polarization of individual researchers and groups. Zhang pointed out that to avoid the information cocooning phenomenon, researchers should be committed to breaking the established cognitive framework, constantly breaking through cognitive boundaries, and having problem awareness and rootedness in research methods (Zhang Y. H., 2020).

The key to solving the problem of information cocoon for researchers is to focus on the information behavior of researchers and clarify the formation mechanism and operation mechanism of information cocoon researchers. Avoid the adverse effects of information cocoon while meeting the information needs of researchers in the research field, and create a good information environment for researchers.

## 2. Literature review

### 2.1 Researcher information behavior

Information behavior refers to human behavior regarding information sources and channels, including active and passive information retrieval and utilization (Li and Xu, 2022). With the rapid development of information digitization, researchers’ knowledge creativity Zhou et al. pointed out that the information behavior of researchers contains four aspects: information attention behavior, information acquisition behavior, cognitive information behavior, and information release behavior (Zhou and Zou, 2015). Among them, information concern behavior, information acquisition behavior, and information cognition behavior belong to the input of the information by researchers, and information release behavior belongs to the output of information. The output of researchers’ innovative results cannot be achieved without the input of high-quality information in related fields. Therefore, when studying researchers’ information behaviors, we should focus on the behaviors related to information input.

The information-focused behavior of a researcher is the beginning of scientific work. Only when scientific researchers start to pay attention to a certain field can they move to the next stage of information acquisition. In the Internet era, the explosive growth of information makes people suffer from information overload and have to avoid information subjectively to reduce the burden. Scientific research knowledge exchange is an information-intensive interactive activity, and researchers, as the receiver, need to input a large amount of information in a relatively short period of time and inevitably ignore and avoid the knowledge transmitted by the knowledge sharers in order to clarify the scientific research information avoidance behavior of researchers Gong et al. constructed a mechanism framework for the influence of information avoidance behavior in scientific research knowledge exchange (Gong et al., 2022).

The current academic community has paid more attention to the information acquisition behaviors of researchers. The information acquisition behaviors of researchers mainly include data reuse behaviors, collaborative information behaviors, self-storage behaviors, and data-sharing behaviors. For example, in terms of data reuse behavior, Zhang et al. explored the characteristics of data reuse behavior of social science researchers, taking the field of management in China as an example (Zhang et al., 2020). Sun et al. explored the influencing factors of data reuse behavior of social science researchers based on the MOA theoretical framework. They found that the motivation, opportunity, and ability of social science researchers’ data reuse were important influences on their data reuse behaviors (Sun et al., 2021). Regarding collaborative information behavior, Xie et al. analyzed the collaborative information behavior between researchers and disciplinary librarians at different stages by combining life cycle theory and coconstructing collaborative information behavior guarantee systems for scientific data services (Xie et al., 2020). In terms of self-storage behavior, Yuan sorted out relevant studies on researchers’ awareness and recognition of self-storage, the current situation of participation behavior, and influencing factors. The results showed that supporting the concept of free and open access to scientific research results and improving scientific performance is conducive to promoting researchers’ participation in self-storage. In contrast, factors such as copyright disputes hinder researchers’ self-storage behavior (Yuan, 2018). Regarding data-sharing behavior, Zheng used Meta-analysis to explore the factors influencing researchers’ willingness to share data and showed subjective norms. Perceived benefits and perceived ease of use positively enhance researchers’ willingness to share data, while age, perceived costs, and perceived risks play a negative role (Zheng, 2021). In addition, in the current mobile Internet information environment, researchers’ information behavior is characterized by increased network dependence and “fragmentation” of information access, making information encounters an essential mode of information search and discovery. Hu et al. explored the mechanism of researchers’ information encounters from a process perspective with the help of a system dynamics model and emphasized the critical roles of three types of factors, namely, users, information, and context, in the information encounter system of researchers (Hu et al., 2020).

In addition, the cognition of research information is also an essential part of the information input process of researchers. Brenda Dervin proposed in the meaning construction theory that “information is the result of human understanding (Dervin, 1998).” Research innovation originates from thinking about and reconstructing information (Bailey and Tierney, 2002), and researchers’ information output behavior depends on the input information’s understanding and cognition.

It is worth noting that many scholars have pointed out the negative effect of the explosive growth of information in the Internet era, i.e., information overload (Fuertes et al., 2020; Gong et al., 2022). The sheer volume of information overwhelms researchers. Researchers may subconsciously or even actively ignore other information that is not relevant to the current research but is valuable. If researchers focus only on their familiar research areas for a long time and ignore other valuable information intentionally or unintentionally, it may eventually lead to the information cocoon effect. In recent years, some scholars have also paid attention to this problem, such as Yuan, who used the Grounded Theory research method to conduct a qualitative study on the factors influencing the formation of researchers’ information cocoon in the network environment through interviews, data coding, and model construction (Yuan, 2018). Although his study explains to a certain extent how the information cocoons of researchers are generated, the research form of interviewing and coding has an intense subjectivity, which makes different coders may get different conclusions. Therefore, quantitative research can remedy this deficiency to a certain extent.

In summary, it is clear that researchers are at the forefront of the development of the times, and the process of their information input has an important impact on the output of scientific research results and therefore has received wide attention from the academic community. In the era of data overload, it is clear that the information cocoon effect has had an impact on the lives of the general public and also on the information behavior of researchers. In order to avoid the cognitive pressure brought by information overload, researchers often choose to activate their filtering mechanism to reject information that does not seem relevant to their research, which may lead to excessive homogenization of the acquired information and gradually wrap themselves in the information cocoon. The research on the formation mechanism of the information cocoon effect of researchers is urgent. Few scholars have paid attention to the information cocoon of researchers. Only a small number of studies have used qualitative methods to explore the causes of the information cocoon of researchers. However, the results are subjective, and there is an urgent need to use a more scientific and objective method to There is an urgent need to use a more scientific and objective method to explore further the issue of the information cocoon effect of researchers.

### 2.2 Information cocoon effect

Keith Sunstein, a professor at Harvard University, first proposed the concept of the information cocoon in 2006, arguing that because the public only focuses on information that interests them, in the long run, they will shackle themselves in the cocoon-like a silkworm chrysalis, i.e., the information cocoon (Sunstein et al., 2008). Faced with a considerable amount of information, human attention is limited. When unable to handle the overload of information, they often engage selectively based on their interests and filter and select information based on their personal preferences to keep their attention focused and psychologically comfortable (Zhao, 2021). The development of algorithmic recommendation technology has provided the public with more personalized information services. However, because the public’s own information needs are not comprehensive, they often choose to pay attention to their favorite things or information areas that can make them happy and are not willing to actively focus on other information, which will limit the breadth and depth of their access to information in the long run (Ren et al., 2021). Users tend to isolate themselves in environments of homogenous and like-minded communication and are more eager to become friends with those who have similar opinions, which might increase internal group identification and devalue external groups (Cargnino and Neubaum, 2020).

The information cocoon has become a hotspot in informatics-related fields in recent years. Scholars mainly research the causes, influencing factors, and dissipation strategies of information cocoon from the perspectives of algorithmic technology, social interaction, and human factors characteristics. For example, in terms of algorithmic techniques, Liu et al. combined multi-label classification algorithms with building a multidimensional feature labeling system to cope with the information cocooning problem (Liu et al., 2022). In terms of social interaction and human factors features, Baumann et al. qualitatively reproduced the observation relationship between user participation and opinions, as well as the opinion isolation in the interactive network by building a dynamic model, revealing the core mechanism of echo chamber and polarization in social media (Baumann et al., 2020); Zhang combined S-O-R theory to explore the factors influencing users’ information echo behavior in the big data environment and found that users’ affective and cognitive factors have a more significant influence on users’ information echo behavior. Their internal motivation and external stimuli indirectly influence information echo behavior through changes in cognition (Zhang H., 2020). Zhang et al. looked at information overload, selective contact psychology and adverse effects of technology, etc., explored the causes and their effects of the college student group caught in the information cocoon, and proposed a path for information literacy education in colleges and universities to dissipate the information cocoon (Zhang et al., 2021).

Whether the information cocoon effect is more beneficial than detrimental or more detrimental than beneficial is not a consistent view in academia. Zhou pointed out that the information cocoon has two sides: on the one hand, the information cocoon makes the audience information-bound; on the other hand, it is a conscious choice of the audience in the case of information overload, and although the information cocoon can be a potential threat to the cohesion of social consensus, it has a specific positive role in the process of reaching social consensus (Zhou, 2019). However, most scholars believe that the adverse effects of the information cocoon effect are severe and difficult to control. For example, Chen et al. analyzed the Lianyungang anti-nuclear incident. They found that the information cocoon induces the formation and fusion of group identity and in-group polarization, leading to the generation of neighborhood avoidance cluster behavior (Chen et al., 2021). A considerable number of scholars analyzed the possible adverse effects of information cocoons. It proposed corresponding countermeasures to dissipate them, such as Shen, who argued that information cocoons could cause cognitive differences among audiences and groups, which may cause the problem of social tearing in the long run, and gave countermeasures to dissipate information cocoons and promote information fairness from different perspectives, such as information platforms and governments (Shen, 2020). Zhang pointed out that once the information cocoon becomes a normalized condition in cyberspace, the space for the Party’s voice to spread through the Internet will be suppressed, harming social stability and national ideological security (Zhang L. L., 2021). Social media networks are becoming more and more important in shaping political views and allowing people to access information. The social relevance of the information cocoon theory will only grow. Contact with different views is essential for cultivating well-informed citizens. In contrast, contact with like-minded voices may lead to the extreme polarization of ideology (Bright, 2016).

In summary, most scholars believe that the information cocoon effect does more harm than good. Many adverse effects are difficult to control, so it is essential to understand their mechanism and control their adverse effects. Most existing studies focus on the information cocoon effect of social network users and university students. However, there is a lack of research on the information cocoon effect of researchers. As an important talent resource for the development of national science and technology, scientific researchers’ innovation ability plays an important role in the development of the times. The information behavior of scientific researchers affects the output of their innovative achievements. Homogeneous scientific research information may be conducive to the researchers’ in-depth understanding of a problem in the short term, but if they get similar information for a long time to avoid information overload, they may fall into the information cocoon. Therefore, this study will investigate the formation mechanism of the information cocoon effect on researchers and the main reasons that influence the formation of an information cocoon. In addition, we want to know what types of researchers are more likely to be affected by the information cocoon.

## 3. Theoretical basis and research hypothesis

Information ecology theory was first proposed by F. W. Horton, a famous scholar in the field of information management, in 1978, which explains the process of information dissemination and diffusion from the perspective of the ecosystem (Yang et al., 2017). The theory has good explanatory power and applicability in information resource management and user information behavior. Lippitt et al. pointed out that individual characteristics and environmental characteristics are important factors affecting human behavior (Lippitt, 1939; Wang et al., 2022).Wang et al. pointed out that information, information person, information technology, and information environment are essential factors that make up the information ecosystem (Wang et al., 2018), in which the information person is the subject and dominates the whole process of information activities. Information is the object and does not depend on human will; information technology is the carrier of information dissemination. The information environment is where the information person as the subject and information as the object interact (Jiang et al., 2020). The information behavior of information people and the interaction between them and other elements are the critical issues studied by this theory, its own filtering mechanism may have a considerable impact on its information preference (Bruns, 2017).

**Figure 1:**
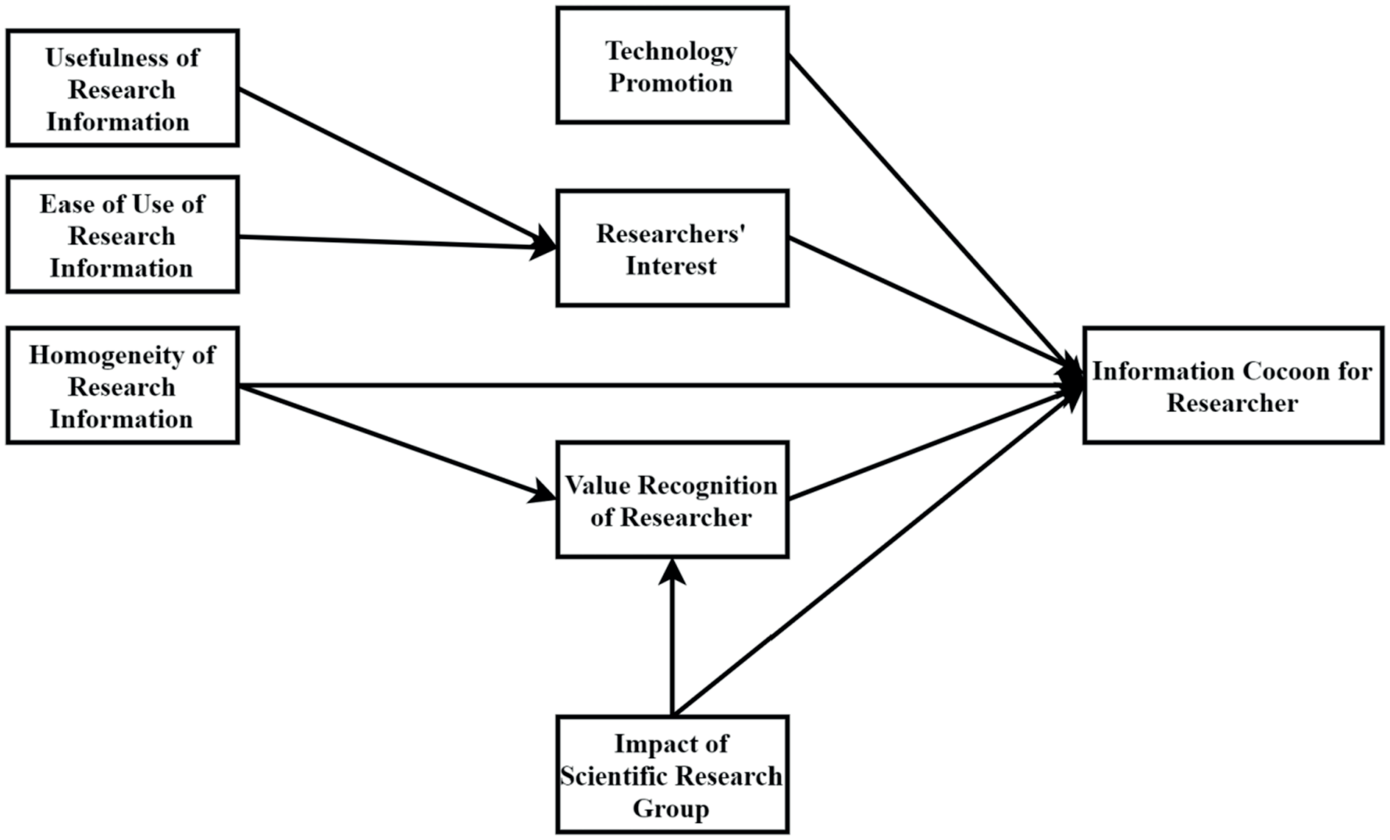
Research model.

Information ecology theory has been widely used in many disciplines, such as management, library, and intelligence science. Based on information ecology theory, Duan Aloe et al. explored the formation mechanism of the information cocoon of Internet users and the operation mechanism. They found that the cognition of value users, information environment, and information technology factors are important influencing factors that affect the formation of the information cocoon of Internet users (Duan et al., 2020). Zhang conducted a qualitative study on the formation mechanism of network users’ information cocoons using rooting theory. Moreover, the results showed that the human information factor, information factor, information technology factor, and information environment factor are essential factors in forming network users’ information cocoons (Zhang H., 2021). This shows that it is applicable and feasible to apply the information ecology theory to explain the formation mechanism of the information cocoon of researchers. Based on the information ecology theory and the characteristics of researchers’ information behavior, this study constructs a research model of the formation mechanism of researchers’ information cocoons, as shown in Figure 1.

Information people are the core elements in the information ecology, in which the producers, processors, transmitters, and users of information all belong to information people, and the object of this study is university researchers, i.e., users of research information. Many scholars believe (Jiang et al., 2020; Zhang H., 2020) that users’ information behavior has an essential connection with their interests and value recognition. Researcher’s interest reflects the degree of enthusiasm and enjoyment in pursuing the needs of the research field. But value recognition of researcher reflects the values formed by the researcher’s recognition of certain types of values. Specifically, when researchers read information related to their research field, they will be highly interested in reading and have pleasant emotions. The emotional pleasure will motivate them to continue to pay attention to the academic information they are interested in. From the perspective of rational cognition, the values conveyed by the information researchers receive are often expected to be highly compatible with the values they have accumulated in the past. Moreover, receiving academic information with highly similar values for a long time will often lead to the formation of value recognition, which will motivate researchers to continue to pay attention to homogeneous scientific information and may even resist academic information that contradicts their views but is meaningful.

*H1:* Researcher’s interest and value recognition significantly influence the formation of information cocoons of researchers.

The information factor is the central system in the information ecology, which is the critical link to other factors in the information ecology. In forming information cocoons, researchers’ exposure to academic information tends to exhibit characteristics such as usefulness, ease of use, and homogeneity. In this study, usefulness of research information refers to the extent to which research-related information improves the performance of researchers and enhances the quality of their research. Researchers tend to show great interest in the information they receive when it is relevant to their research field and beneficial to improving the quality of their research and learning about the details of the information promptly. Ease of use of research information refers to the ease of access to academic information. Information that is easy to access and use is more likely to be downloaded and read by researchers. Homogeneity of research information refers to the degree of homogeneity of research information obtained by researchers, precisely the convergence of research-related information content, access, and the value of the information conveyed. When researchers read academic information related to their research fields or similar to their views, it can often stimulate their interest in reading and effectively improve their research efficiency. However, suppose researchers read information with similar content and viewpoints through fixed information access channels for a long time. In that case, their values are likely to be highly convergent with the transmitted information and form value identities, thus limiting the emergence of other heterogeneous information. In the long run, researchers will be enclosed in such highly similar information, which will be detrimental to their research innovation and may eventually lead to the emergence of the information cocoon effect, based on which the following hypothesis is proposed.

*H2:* The usefulness of research information and ease of use significantly positively affect researchers' interest.

*H3:* Homogeneity of research information has a significant positive impact on the value recognition of researcher and the formation of an information cocoon.

Information technology is a guaranteed factor for the proper functioning of information ecology, which mainly includes information retrieval, processing, dissemination, and security technologies. This study considers technology promotion as the information technology factor, i.e., the subjective feelings of researchers about each service function, each service module, and each application technology of the research service platform. Compared with the past, modern information technology has made it possible for researchers to access relevant academic information and use the academic platform more conveniently. The convenience brought by information technology should promote access to diversified research information. However, at the same time, the complicated and overloaded information also makes it more difficult for researchers to obtain and identify useful information. In order to avoid this situation, researchers often focus only on specific research topics, research teams, and keywords or choose a fixed and usual platform to obtain relevant academic information. The following hypothesis is proposed based on the fact that the information content and access will be solidified and thus become dependent on the information, thus intensifying the formation of the information cocoon effect of researchers.

*H4:* Technology promotion has a significant positive impact on the formation of an information cocoon for researchers.

The information environment is an essential condition for the normal functioning of the information ecology, including the internal and external environments, including the social environment, impact of scientific research group, and opinion leaders. For researchers, the academic fields they can cover are mainly influenced by their research teams. Generally speaking, researchers in the same research team have a high degree of similarity in their research contents and fields, which are shared through internal teamwork and academic group meetings. Similar academic information is disseminated for a long time, which may lead to narrowing information, group polarization, and even the information cocoon effect among researchers.

*H5:* Impact of scientific research group has a significant positive impact on the value recognition of researcher and the formation of information cocoons.

## 4. Research design

### 4.1 Questionnaire

The questionnaire consists of three parts: the first part introduces the basic information of the questionnaire, including the purpose and content of the survey; the second part investigates the basic information of the respondents, including demographically important information such as gender, age, highest education, discipline, and the number of research teams. The third part is the investigation of the factors influencing the formation mechanism of the information cocoon of researchers, including researcher’s interest, value recognition of researcher, usefulness of research information, ease of use of research information, homogeneity of research information, technology promotion, and impact of scientific research group. Moreover, the information cocoon of researchers’ eight variables and variable items are from the existing literature of mature scales, with appropriate adaptations in the study context and appropriate amendments based on the pre-research part. The items were measured on a 5-point Likert scale.

### 4.2 Questionnaire distribution and data collection

The research subjects of this study were university researchers, and the questionnaires were distributed by a combination of online and offline methods. Three hundred and thirty-four questionnaires were collected, and 320 valid questionnaires were obtained after excluding those that did not answer the screening questions correctly, those that took less time to fill in, and those with too high a repetition rate.

## 5. Data analysis

### 5.1 Descriptive analysis

In the formal research, the number of males was 156, accounting for 51%. The number of females was 164, accounting for 49%, with a relatively balanced gender distribution. in terms of age distribution, researchers aged 21–30 accounted for the most significant proportion, about 50%, followed by researchers aged 31–40, about 39%. Furthermore, this indicates that young and middle-aged researchers are the leading group in this research. Regarding the distribution of research disciplines, science and engineering accounted for the most significant proportion, about 20%, followed by management and education, about 14%. In terms of education, most of the researchers in this study had the highest degree of master’s degree, about 76%, and doctoral degrees only accounted for 24%; in terms of the status of researchers, the proportion of students and teachers was the same. This indicates that this research sample meets this study’s needs.

### 5.2 Reliability testing

As shown in Table 1, Cronbach’s α coefficients of all the variables in the questionnaire are more significant than 0.7. Cronbach’s α coefficient does not increase again after excluding any measure, and the scale’s reliability is good. The tests of structural validity include convergent validity and discriminant validity. The factor loadings, combined reliability (CR), and average extracted variance (AVE) can verify the convergent validity. The factor loadings of each item are higher than 0.7, the CR is higher than 0.8, and the average extracted variance (AVE) is higher than 0.5. The discriminant validity can be verified by the square root of the observed variable AVE value more significant than the correlation coefficient with other variables. As shown in Table 2, the square root of all variable AVE values is more significant than the correlation coefficients with other variables, which shows that the questionnaire has good discriminant validity.

**Table 1:** Reliability testing.

Variables	Measure	Cronbach’s alpha coefficient	Cronbach’s alpha coefficient after removal of the measure	Factor loadings	CR	AVE
Researcher’s interest	PI1	0.707	0.572	0.821	0.837	0.632
	PI2		0.647	0.776		
	PI3		0.630	0.787		
Value recognition of researcher	PV1	0.774	0.716	0.821	0.871	0.692
	PV2		0.752	0.796		
	PV3		0.615	0.876		
Usefulness of research information	IU1	0.751	0.700	0.746	0.836	0.505
	IU2		0.727	0.642		
	IU3		0.712	0.716		
	IU4		0.699	0.731		
	IU5		0.699	0.714		
Ease of use of research information	IE1	0.777	0.712	0.827	0.872	0.694
	IE2		0.736	0.811		
	IE3		0.650	0.861		
Homogeneity of research information	IS1	0.878	0.823	0.902	0.926	0.806
	IS2		0.875	0.866		
	IS3		0.781	0.924		
Technology promotion	TE1	0.729	0.639	0.801	0.834	0.557
	TE2		0.714	0.661		
	TE3		0.666	0.763		
	TE4		0.655	0.754		
Impact of scientific research group	EV1	0.853	0.725	0.920	0.912	0.776
	EV2		0.820	0.867		
	EV3		0.837	0.854		
Information cocoon for researcher	CO1	0.891	0.839	0.909	0.932	0.821
	CO2		0.854	0.900		
	CO3		0.840	0.909		

**Table 2:** Distinct validity test.

	RI	VR	UI	EI	HI	TP	RG	CO
RI	**0.795**							
VR	0.379**	**0.832**						
UI	0.419**	0.248**	**0.712**					
EI	0.178**	0.316**	0.388**	**0.833**				
HI	0.162**	0.331**	0.124*	0.212**	**0.898**			
TP	0.403**	0.331**	0.524**	0.071	0.106	**0.746**		
RG	0.198**	0.347**	0.226**	0.230**	0.209**	0.284**	**0.881**	
CO	0.097	0.409**	0.010	0.332**	0.437**	0.014	0.411**	**0.906**

RI = researcher’s interest, RV = value recognition of researcher, UI = usefulness of research information, EI = ease of use of research information, HI = homogeneity of research information, TP = technology promotion, RG = impact of scientific research group, CO = information cocoon for researchers.

** At the 0.01 level (two-tailed), the correlation is significant.

* At the 0.05 level (two-tailed), the correlation is significant. The bold values are the square root of each variable AVE.

### 5.3 Individual variation analysis

From the results of the independent sample t-test in Table 3, it can be seen that the differences between genders in usefulness of research information, homogeneity of research information, technology promotion, impact of scientific research group, and information cocoon of researcher are significant. The mean values of usefulness of research information and technology promotion for female researchers were significantly higher than those for male researchers. The mean values of homogeneity of research information, impact of scientific research group, and researcher information cocoon for male researchers were significantly higher than those for female researchers.

**Table 3:** Differences in the dimensions among gender-specific researchers.

Dimension	P	T	Standard deviation	Average	Gender
Researcher’s Interest	0.592	0.536	0.463	4.376	Female
			0.533	4.346	Male
Value recognition of researcher	0.126	−1.535	0.716	4.246	Female
			0.387	4.344	Male
Usefulness of research information	0.006	2.777	0.293	4.373	Female
			0.508	4.244	Male
Ease of use of research information	0.830	−0.214	0.669	4.000	Female
			0.753	4.017	Male
Homogeneity of research information	0.006	−2.763	0.941	3.644	Female
			0.716	3.902	Male
Technology promotion	0.037	2.095	0.392	4.372	Female
			0.527	4.263	Male
Impact of scientific research group	0.000	−4.240	0.815	3.839	Female
			0.437	4.148	Male
Information cocoon for researcher	0.004	−2.879	0.849	3.848	Female
			0.601	4.083	Male

The independent sample *t*-test in Table 4 shows significant differences in value recognition of researcher, ease of use of research information, impact of scientific research group, and the information cocoon of researcher. The mean values of value recognition of researcher, ease of use of research information, impact of scientific research group, and information cocooning of researchers were significantly higher for researchers with the highest degree of PhD than for those with the highest degree of master’s. This indicates that higher-education researchers are more likely to be affected by the information cocoon effect.

**Table 4:** Differences in dimensions among researchers with different degrees.

Dimension	P	T	Standard deviation	Average	Academic qualifications
Researcher’s interest	0.200	−1.285	0.503	4.342	Master
			0.476	4.425	PhD
Value recognition of researcher	0.015	−2.434	0.592	4.250	Master
			0.519	4.434	PhD
Usefulness of research information	0.763	0.302	0.402	4.314	Master
			0.461	4.297	PhD
Ease of use of research information	0.000	−5.933	0.767	3.918	Master
			0.359	4.298	PhD
Homogeneity of research information	0.699	−0.387	0.850	3.760	Master
			0.844	3.802	PhD
Technology promotion	0.612	0.508	0.407	4.328	Master
			0.618	4.290	PhD
Impact of scientific research group	0.034	−2.136	0.702	3.949	Master
			0.568	4.118	PhD
Information cocoon for researcher	0.002	−3.124	0.780	3.900	Master
			0.588	4.162	PhD

From the results of the independent sample *t*-test in Table 5, it is clear that the differences in value recognition of researcher, homogeneity of research information, and information cocoon of researchers between researchers as teachers and students are significant. Among the researchers, the mean value recognition of researcher of teachers is significantly higher than that of students. In contrast, the mean value of students is significantly higher than that of teachers in terms of homogeneity of research information and the information cocoon of researchers.

**Table 5:** Differences in dimensions among researchers with different roles.

Dimension	P	T	Standard deviation	Average	Roles
Researcher’s interest	0.206	−1.269	0.624	4.325	Student
			0.333	4.340	Teacher
Value recognition of researcher	0.031	−2.162	0.626	4.222	Student
			0.527	4.361	Teacher
Usefulness of research information	0.189	−1.317	0.515	4.278	Student
			0.292	4.340	Teacher
Ease of use of research information	0.140	1.478	0.707	4.068	Student
			0.710	3.951	Teacher
Homogeneity of research information	0.001	3.416	0.674	3.932	Student
			0.961	3.616	Teacher
Technology promotion	0.111	−1.598	0.587	4.276	Student
			0.304	4.360	Teacher
Impact of scientific research group	0.170	1.375	0.629	4.043	Student
			0.715	3.939	Teacher
Information cocoon for researcher	0.092	1.690	0.651	4.034	Student
			0.824	3.894	Teacher

As shown in Table 6, there are significant differences in the perceptions of information usefulness, information ease, homogeneity of research information, technology promotion, impact of scientific research group, and researcher information cocoon among researchers of different ages. The comparison shows that researchers aged 51 and above have the highest mean values in information usefulness, information ease of use, homogeneity of research information, and researchers’ information cocoon. Moreover, the mean values of researchers’ information cocoon show a wave-like increase with age; researchers aged 31–40 have the highest mean values in technology promotion and impact of scientific research group.

**Table 6:** Differences in the dimensions among researchers of different ages.

Dimension		Researcher’s interest	Value recognition of researcher	Usefulness of research information	Ease of use of research information	Homogeneity of research information	Technology promotion	Impact of scientific research group	Information cocoon for researcher
P		0.075	0.427	0.004	0.000	0.000	0.001	0.000	0.000
F		2.320	0.929	4.611	8.611	8.317	6.049	10.979	9.639
Under 30	SD	0.563	0.667	0.349	0.594	0.810	0.577	0.677	0.789
	AVE	4.317	4.250	4.341	4.067	3.867	4.275	3.917	3.927
31–40	SD	0.434	0.466	0.475	0.688	0.843	0.211	0.582	0.541
	AVE	4.449	4.358	4.329	4.065	3.755	4.436	4.210	4.132
41–50	SD	0.325	0.576	0.422	1.125	0.846	0.485	0.626	0.983
	AVE	4.238	4.298	4.036	3.381	3.107	4.071	3.583	3.345
Over 51	SD	0.356	0.178	0.396	0.178	0.415	0.267	0.975	0.502
	AVE	4.333	4.167	4.350	4.167	4.375	4.250	3.458	4.208

Table 7 shows significant differences in the perceptions of value recognition of researcher, information ease, homogeneity of research information, technology promotion, impact of scientific research group, and researcher information cocoon among researchers of different team sizes. The mean values of value recognition of researcher, ease of use, homogeneity of research information, technology promotion, impact of scientific research group, and information cocoon of research teams of 20 or more people are greater than those of research teams of other sizes.

**Table 7:** Differences in dimensions among researchers of different sizes of research teams.

Dimension		Researcher’s interest	Value recognition of researcher	Usefulness of research information	Ease of use of research information	Homogeneity of research information	Technology promotion	Impact of scientific research group	Information cocoon for researcher
P		0.197	0.000	0.092	0.022	0.002	0.017	0.014	0.004
F		1.517	5.997	2.015	2.892	4.255	3.046	3.157	3.925
5 and below	SD	0.622	0.656	0.274	0.529	0.423	0.211	0.403	0.158
	AVE	4.167	4.000	4.250	3.917	3.823	4.188	3.781	4.010
6–10	SD	0.510	0.711	0.287	0.751	0.906	0.412	0.624	0.901
	AVE	4.405	4.212	4.369	3.924	3.748	4.343	4.048	3.786
11–15	SD	0.572	0.395	0.671	0.863	0.889	0.663	0.796	0.846
	AVE	4.370	4.375	4.217	4.019	3.787	4.236	3.907	4.106
16–20	SD	0.351	0.291	0.430	0.590	0.926	0.471	0.884	0.304
	AVE	4.364	4.424	4.277	4.061	3.439	4.318	3.879	4.076
20 or more	SD	0.158	0.224	0.090	0.112	0.392	0.152	0.269	0.164
	AVE	4.344	4.583	4.369	4.375	4.229	4.531	4.281	4.208

### 5.4 Model hypothesis testing

The model path coefficients were tested against the hypotheses, and the results are shown in Table 8. From the test results, it can be seen that value recognition of researcher (*β* = 0.277, *p* < 0.001), homogeneity of research information (*β* = 0.307, *p* < 0.001), and impact of scientific research group (*β* = 0.311, *p* < 0.001) have a more significant positive effect on the formation of researchers’ information cocoons. The positive effect of impact of scientific research group was the most pronounced, but the role of technology promotion and interest in forming researchers’ information cocoons was not verified. Information usefulness (*β* = 0.412, *p* < 0.001) significantly positively affected researchers’ interest, and its role in influencing interest was not verified. Both homogeneity of research information (*β* = 0.270, *p* < 0.001) and impact of scientific research group (*β* = 0.291, *p* < 0.001) had a significant positive effect on value recognition of researcher. Moreover, impact of scientific research group has a slightly higher effect than homogeneity of research information.

**Table 8:** Path coefficients and hypothesis testing results.

NUM	Independent variable → dependent variable	Standardized path coefficient	Standard error	T	P	Validation results
1	RI → CO	−0.047	0.077	−0.921	0.358	Not supported
2	VR → CO	0.277	0.068	5.200	0.000	Supported
3	UI → RI	0.412	0.066	7.441	0.000	Supported
4	EI → RI	0.018	0.039	0.329	0.743	Not supported
5	HI → VR	0.270	0.035	5.217	0.000	Supported
6	HI → CO	0.307	0.042	6.409	0.000	Supported
7	TP → CO	−0.179	0.082	−3.519	0.000	Not supported
8	RG → VR	0.291	0.044	5.623	0.000	Supported
9	RG → CO	0.311	0.054	6.339	0.000	Supported

In academic research, researchers tend to collect and obtain academic information on research topics and methods similar to their field. This type of information is more likely to stimulate their interest in reading. Although the ease of use of information brings convenience to researchers’ research, it is not enough to stimulate researchers’ interest in reading. The influence of interest on the information cocoon of researchers is not significant, probably because interest only reflects the shallow emotional state of researchers, and interest in a specific field of research may influence other information retrieval behavior. However, its influence does not yet lead to the formation of the information cocoon of researchers. Value recognition of researcher reflects the deeper cognitive state of researchers. Homogeneity of research information and value recognition of researcher play a positive role in forming researchers’ information cocoon. Long-term input of homogeneous information may cause researchers to develop a strong value recognition of researcher for a particular viewpoint. In the short term, access to a large amount of academic information in the same field and on the same topic can help researchers gain a deeper understanding of that particular field. However, in the long term, solidifying information sources and content may narrow information for researchers. Homogenized information will change the value perception of researchers and lead to the solidification of thinking, which may eventually lead to the formation of information cocoons for researchers, which not only affects their academic careers but also is not conducive to the prosperity of the academic community. For researchers, the most frequent social group they come into contact with is their research group. The convergence of academic topics within the group, the fixed academic communication groups, and a large amount of homogeneous academic information increase the possibility of polarization of the research community and the degree of scientific involution. At the information technology level, the correlation between technology promotion and the information cocoon of researchers is insignificant, indicating that technology’s development does not impact researchers’ formation. To circumvent researchers’ information cocoon effect, we should consider the influence of information homogenization, researchers themselves, and environmental factors.

## 6. Discussion

### 6.1 Comparison with previous research

In the past, researchers’ information behavior has been deeply discussed. However, with the development of Internet technology, the amount of information available to people has shown an explosive growth trend. Researchers have encountered a new problem: how to extricate themselves from complex information. Some researchers have paid attention to the possible information avoidance behavior of researchers. Researchers may intentionally or unintentionally start their information filtering mechanism, selectively focus on information related to their research, and ignore other seemingly unrelated information, even if the ignored information is valuable. Avoiding information may reduce the pressure on researchers and temporarily remove them from the confusion and negative emotions existing in current research. Nevertheless, it may also cause researchers to ignore important information and miss innovative ideas and opportunities to eliminate uncertainty (Keren, 2018). Selective information input may lead to information showing a high degree of homogeneity, bringing risks to the information cocoon.

In order to deal with the problem of researchers’ information cocoon, this study used quantitative methods to build a theoretical model of the formation mechanism of scientific researchers’ information cocoon and made a detailed analysis of the differences between different scientific researchers’ formation of a research information cocoon.

When discussing the effect of information cocoons, previous scholars chose social network users as their research objects. In today’s era, researchers are also affected by information overload. This study found that the influence of scientific research groups, homogeneity of research information, and value recognition of researcher are important factors affecting the formation of an information cocoon for scientific researchers. Yang et al. analyzed the causes and consequences of the information cocoon, pointing out that different environments will affect personal information contact behavior and adjust the impact of personal characteristics on the information cocoon (Yang and Yuan, 2022). Their research conclusion is consistent with that of this study.

In terms of information homogeneity, Zhang et al. analyzed the influencing factors of network users’ information cocoon detention willingness based on PPM theory. They found that information homogeneity and information overload negatively affect network users’ information cocoon detention willingness (Zhang and Li, 2022). However, this study found that information homogeneity positively impacts the generation of an information cocoon. The different research objects may be an important reason for this difference. Social network users are eager to obtain more extensive information through social network platforms. A large amount of homogenized information for a long time will put users in a state of overload, which is easy to generate negative emotions such as fatigue and boredom, thus generating the desire to leave the original information cocoon. However, for researchers, a deep understanding of their fields can not be separated from reading a large number of relevant literature. Therefore, compared with network users, scientific researchers’ information cocoon effect is more difficult to control.

In value recognition, Yu et al. pointed out that the information cocoon presents the coexistence of tool rationality and value rationality. That is, improving the subjective initiative of the audience and high-dimension media literacy has positive value for overcoming the information cocoon (Yu and Wang, 2022). Their conclusion suggests that although the value recognition of scientific researchers may further promote the formation of the information cocoon, it is not absolute. Researchers should give play to their subjective initiative, take the initiative to overcome their absolute identification with a single point of view or specific areas, and be more inclusive of multiple views.

We initially believe that the information cocoon of scientific researchers will also be positively affected by research interest, technology, and other factors. However, the relationship between interest and information cocoon has not been confirmed. Much research on network users emphasizes the critical influence of users’ interests on the information cocoon. For example, Ren et al. pointed out that individual selective psychology determines the evolution of their information behavior, constraining users to narrow information cocoons guided by their existing ideas and interests, and this phenomenon is objective (Ren et al., 2021). However, some scholars pointed out that users only have the information cocoon effect when the values of users are deeply affected or even changed in the reading process. The value cognition of users, rather than the emotional cognition represented by interest, is the critical factor in the formation of the information cocoon effect (Duan et al., 2020). Researchers should improve their own information literacy, actively focus on information in different fields, and embrace different values. In this study, the relationship between technology promotion and information cocoon is another untested hypothesis. The research results show that technology promotion does not accelerate the generation of information cocoon for scientific researchers but harms this process. Some scholars pointed out that the influence of information technology on the information cocoon effect is weaker than that of the human information element (Yan and Li, 2022). Technology is a double-edged sword. Although many scholars pointed out that the development of information technology may produce a series of adverse effects, including information overload, we cannot deny that technology provides us with more information access channels. We should pay more attention to the role of people themselves.

As the characteristics and status of the information behavior subject, the human information factor is a primary essential category in forming the user information cocoon. It is necessary to analyze the individual differences in forming an information cocoon. Previous studies focused on the information cocoon’s causes, hazards, and elimination strategies. Compared with previous studies, this study further explored the differences between different types of individuals based on path analysis. The research found that the different gender, educational backgrounds, identities, ages, and team sizes of researchers will lead to different dimensions in producing the information cocoon.

### 6.2 Differences among different types of researchers

The mean values of female researchers were significantly higher than those of male researchers in terms of perceived information usefulness and technology promotion. In comparison, the mean values of male researchers were significantly higher than those of female researchers in terms of information homogeneity, impact of scientific research group, and researcher information cocoon. The subsequent path analysis showed that information homogeneity and impact of scientific research group significantly positively affected forming of a researcher information cocoon. The results suggest, to some extent, that male researchers are more likely to be trapped in information cocoons. Alrashidi notes that while society is working toward professional equality between men and women, fewer women are actively engaged in research (Alrashidi, 2017), and even for those who are, a significant proportion of them need to allocate a significant amount of time to family activities. As a result, the depth of research, participation, and even academic status of current female researchers is less likely to be on par with that of male researchers. It should be noted that the premise of the information cocoon effect for researchers is the long-term input of a large amount of homogenized information, so female researchers are currently at less potential risk of being threatened by the information cocoon compared to men. However, with the development of society, the gender gap in the research field will continue to narrow, and female researchers should not ignore the adverse effects of information cocooning.

Researchers with the highest degree of PhD have significantly higher mean values of value recognition, information ease, impact of scientific research group, and researcher information cocoon than those with the highest degree of M.S. Higher education also brings a higher risk of an information cocoon. Previously, it was believed that the longer an individual spends on conscious information acquisition, the wider the boundaries of his or her personal information world and the more information-rich the individual is (Zhou and Bao, 2021). However, this study reveals the opposite conclusion, which may be since, compared to master’s degrees, PhDs have developed a more fixed pattern of research work. There is a clear bias in the content and channels of acquiring research information (Spezi, 2016). While having rich research experience, PhDs are also more influenced by the research community than master’s degrees. The content of the information exchanged within the group tends to be the same. The long-term and repeated exchanges within the group will consolidate the researchers’ identification with their views, leading to an increased possibility of information cocooning.

The mean value of mentors was significantly higher than that of students in terms of value recognition of researcher. However, the mean value of students was significantly higher than that of mentors in terms of information homogeneity and information cocooning of researchers. The fact that mentors have richer research experience and have developed more fixed research protocols in continuous exploration may be an important reason why researchers with mentorship status have higher value recognition of researcher than student research groups. However, it is also found that the degree of access to homogeneous information and the risk of information cocooning are higher for student researchers than for mentors. This may be because mentors involved in research have a broader experience and communication platform than students. Students are still immature in research thinking, self-control and drive, and their tendency to avoid information in the exchange of research knowledge is more significant (Gong et al., 2022). Therefore, in research work, mentors should pay more attention to cultivating students and guide them in various forms to face diversified and cross-disciplinary research information with a more open and inclusive attitude to avoid getting caught in the information cocoon.

In addition, this study also found significant differences in the information cocoon formation process among researchers in different-sized research teams. When setting up a team, it is not better to have a larger team size but to have a team of appropriate size according to the nature of the team’s research area to avoid the information cocoon effect. The question of how to allocate team members in different research fields to avoid the research information cocoon effectively is worthy of more in-depth exploration in the future.

### 6.3 Analysis of the formation path of researchers’ information cocoons

It was found that researchers’ interest in reading would be influenced only by the usefulness of the information and not by the ease of use of the information. However, the effect of interest on researchers’ information cocoon was insignificant. This is easy to understand because once a researcher identifies a research direction, his or her subsequent work will inevitably revolve around this precise topic, and helpful information related to the research content is more likely to stimulate the researcher’s interest in reading, regardless of its accessibility. However, it should be clear that the interest of researchers only has a subtle influence on their information acquisition behavior at a superficial emotional level and does not lead to the formation of an information cocoon effect for they. Researchers gradually form their research logic and style in their scientific work, and the concepts and methods conveyed by the information they acquire overlap highly with their previous perceptions. Once researchers are surrounded by similar information, they tend to form a valued identity. This value recognition of researcher will motivate researchers to focus on homogeneous information and may even resist other different but meaningful information (Chen and Wang, 2019).

Both information homogeneity and value recognition of researcher play a positive role in forming researchers’ information cocoons. Homogenized information and solidified values may lead researchers into an information cocoon, where they cannot be thoughtful because their preconceptions will become ingrained, gradually blocking them from outside voices. In Sunstein’s view, people are trapped in information cocoons because they select and filter information (Sunstein et al., 2008). Previously, scholars have focused on the information cocoon effect of Internet users. They believe that the “filtering bubble” of Internet users is generated by information filtering and information pushing based on the information traces of users. However, for researchers， their “filter bubble” is more based on personal choice. In the field of education, there is a remarkable phenomenon: students’ knowledge gradually takes on a pyramidal shape as their education level increases, i.e., the higher the education level, the narrower the field and the more specialized the content they learn. This may seem a paradox because, in the past perception, the highly educated group had a broader knowledge. Higher education approximates to equal broader knowledge in terms of learning and understanding of common sense knowledge. However, in scientific problems, researchers need to conduct an in-depth analysis of specific and concrete problems, which requires them to collect a large amount of relevant research literature actively. It is worth mentioning that these kinds of literature often have strong similarities regarding research content, research methods, and even the expression of opinions. In this process, researchers’ values are constantly crystallized, which is likely to intensify the information narrowing of researchers and eventually lead to the development of the information cocoon to a deeper level.

Communities have a significant positive impact on both the academic value recognition of researcher of researchers and the formation of their information cocoons. Information technology provides a more self-contained space for ideas and vast knowledge in any field. However, while communication within research communities is more efficient, scholarly communication is not necessarily smoother and more effective than in times of information scarcity. Research communities are classed by differentiation and may tend to be homogeneous within groups and heterogeneous between groups. Members within research groups may be limited to their research areas, drastically reducing their communication with other research groups in different fields. Once generated, the information cocoon of scientific researchers may have unpredictable effects on scientific work. Members within a group have similar views and perspectives. Over time, they accumulate their unique styles and characteristics, eventually leading to more significant characteristics of intra-group homogeneity and inter-group heterogeneity. The “spiral of silence theory” proposed by German scholar Neumann suggests that the more silent people are, the more others will consider a particular view representative (Noelle-Neumann, 1974). This theory is often applied to explain the process of spreading online opinions. When people see that more people agree with a specific view, they will participate more actively, reinforcing the view and spreading it to a broader area, and getting more people’s support, which contributes to the homogeneity within the group. This theory can also be used to explain the influence of community on researchers’ academic value recognition and the formation of information cocoons. For example, researchers in the same research group or even in the same research field often communicate with researchers with similar views and research topics and rarely with researchers who hold opposite views or whose research fields are not significantly related. The community brings together researchers with similar research perspectives and fields, strengthening internal value recognition of researcher and reinforcing group members’ research perspectives.

### 6.4 Coping strategies of researchers’ information cocoon

Based on the above analysis, the following strategies and solutions to solve the information cocoon problem of researchers are given.

Researchers of different genders, education, status, age, and team size have different characteristics in the information cocoon effect formation process. Male, highly educated students, and older research groups are more likely to be influenced by crucial variables in information cocoon formation than other researchers. So in scientific research, this person should pay more attention to diversified research contents and methods, promptly follow up on the latest research hot issues, continuously diversify research fields and channels, and avoid homogeneous effects from becoming the dominant sustainable research.

In forming an information cocoon for researchers, impact of scientific research group, information homogeneity, and value recognition of researcher play a significant favorable influence and are inseparable from each other. Information homogeneity and impact of scientific research group positively influence value recognition of researcher, contributing to the formation of researchers’ information cocoons. This suggests that we should pay attention to the input of diversified information in the research process, not limited to the communication within the subject group, but actively promote the positive interaction between subject groups in different fields and cross-disciplines. Moreover, student research groups are easily limited by their thinking in the research process, and the constant input of homogeneous information tends to solidify their thinking. Supervisors should actively guide them to participate in cross-field and multidisciplinary academic conferences to avoid the information cocoon effect effectively.

## 7. Limitations of research

The study may have had problems related to the sample. In the sample for this study, the number of researchers with the highest degree PhD was much smaller than that of researchers with the highest degree or master’s degree. This is to be expected because, in reality, there are far fewer people with PhDs than people with only master’s degrees. It is more difficult to obtain data for this segment. Although those researchers with PhDs are more focused on research, in reality, there are more researchers with master’s degrees, so this data set is still representative to some extent.

Another potential limitation may be that the article only measured subjects quantitatively using a questionnaire. The concept of information cocooning has not been around for a long time. Using only quantitative measures may overlook subjects’ potential information about the information cocoon during the study. However, this study uses a quantitative approach to measure the information cocoon of the researcher, which can clarify more about the ambiguous concept of the information cocoon and has implications for subsequent studies.

## 8. Conclusion and recommendations for future work

This paper analyzes researchers’ information cocoon formation mechanism and their differences based on the information ecology theory and draws the following conclusions. Firstly, the analysis of individual differences reveals that differences in gender, education, status, age, and team size lead to differences in the perception of information cocoons among researchers in the information ecology theory. In addition, in the path analysis, it was found that information homogeneity, value recognition of researcher, and impact of scientific research group are the key factors that lead to the information cocooning of researchers, and the research impact of scientific research group value recognition of researcher.

Future studies could consider selecting a more scientific sample. In addition, more diverse classifications can be considered in terms of individual variability of researchers, e.g., future studies can examine the variability of researchers with different cognitive styles and personality traits in forming information cocoons.

In addition, future research will use more objective and scientific methods to detect the information cocoon effect of researchers in more depth. Some current studies use qualitative methods such as rooting theory to detect the information cocoon effect, which can avoid the constraints brought about by the fixed topic of quantitative methods but are more subjective. The quantitative method in this study can avoid the influence of analysts’ subjective judgment, but there are still certain shortcomings. Future studies can try to combine data mining techniques to obtain the actual information behavior data of researchers to make the conclusions more objective.

## Data availability statement

The original contributions presented in the study are included in the article/supplementary material, further inquiries can be directed to the corresponding author.

## Ethics statement

The studies involving human participants were reviewed and approved by College of Management in Xi’an University of Science and Technology. The patients/participants provided their written informed consent to participate in this study.

## Author contributions

YX contributed to the conception and design of the study and performed the statistical analysis. WC wrote the first draft of the manuscript and wrote sections of the manuscript. All authors contributed to the article and approved the submitted version.

## Conflict of interest

The authors declare that the research was conducted in the absence of any commercial or financial relationships that could be construed as a potential conflict of interest.

## Publisher’s note

All claims expressed in this article are solely those of the authors and do not necessarily represent those of their affiliated organizations, or those of the publisher, the editors and the reviewers. Any product that may be evaluated in this article, or claim that may be made by its manufacturer, is not guaranteed or endorsed by the publisher.
